# Electroactive hydrogel scaffolds for myocardial infarction repair: Integrating MXene, ginseng-derived bioactives, and 3D bioprinting

**DOI:** 10.1016/j.bbiosy.2026.100154

**Published:** 2026-10-29

**Authors:** Azadeh Rejali, Mehdi Ebrahimian-Hosseinabadi, Mohammad Rafienia, Ghasem Dini

**Affiliations:** a Department of Biomedical Engineering, Faculty of Engineering, University of Isfahan, Isfahan, Iran; b Biosensor Research Center, Isfahan University of Medical Sciences, Isfahan, Iran; c Department of Biomaterials, Nanotechnology and Tissue Engineering, School of Advanced Technologies in Medicine, Isfahan University of Medical Sciences, Isfahan, Iran; d Department of Nanotechnology, Faculty of Chemistry, University of Isfahan, Isfahan, Iran

**Keywords:** Myocardial infarction, Electroactive hydrogels, PEG/PCL scaffolds, MXene, Ginseng-derived bioactives, 3D bioprinting

## Abstract

Cardiovascular disease causes substantial mortality. Following myocardial infarction (MI), limited adult myocardial regeneration contributes to adverse ventricular remodeling and heart failure. Ischemic injury causes irreversible cardiomyocyte loss, activates inflammatory pathways including nuclear factor kappa B (NF-κB) and mitogen-activated protein kinase (MAPK) signaling, and increases matrix metalloproteinase (MMP) activity, promoting extracellular matrix remodeling, scar formation, and functional decline. Current therapies improve survival and limit disease progression but do not restore vascularized, mechanically compliant, electrically integrated myocardium. This review critically evaluates electroactive hydrogels for MI repair, focusing on poly(ethylene glycol) (PEG)/poly(ε-caprolactone) (PCL) scaffolds, MXenes, ginseng-derived bioactives, and three-dimensional (3D) bioprinting. PEG/PCL scaffolds offer tunable mechanics, degradation, injectability, and printability; MXenes provide conductive, hydrophilic, hydrogel-compatible interfaces. Long-term MXene performance depends on preserved conductivity in the oxidative and inflammatory post-infarction microenvironment. MXenes are compared with graphene derivatives, carbon nanotubes, metallic nanostructures, and conductive polymers for performance and translation. Ginseng-derived bioactives are examined as potential modulators of oxidative, inflammatory, vascular, and fibrotic processes, emphasizing standardization, controlled release, potency, and multicomponent interactions. 3D bioprinting spatially controls scaffold architecture and localized cue delivery, although structural organization alone does not establish therapeutic efficacy. Together, these technologies define an evidence-informed preclinical cardiac scaffold framework, while integrated-platform efficacy remains unproven. Clinical translation requires comparative validation, long-term in vivo safety assessment, large-animal studies, scalable, reproducible manufacturing, sterilization compatibility, robust quality control, and regulatory strategy.

## Highlights

• Critically benchmarks MXene against major conductive biomaterial classes.
• Distinguishes component-level evidence from the unvalidated full platform.
• Identifies MXene biosafety, electrophysiology, and long-term fate gaps.
• Maps large-animal, clinical, manufacturing, and regulatory readiness.
• Integrates PEG/PCL, MXene, ginseng bioactives, and 3D bioprinting.

## Graphical abstract

**Figure:**
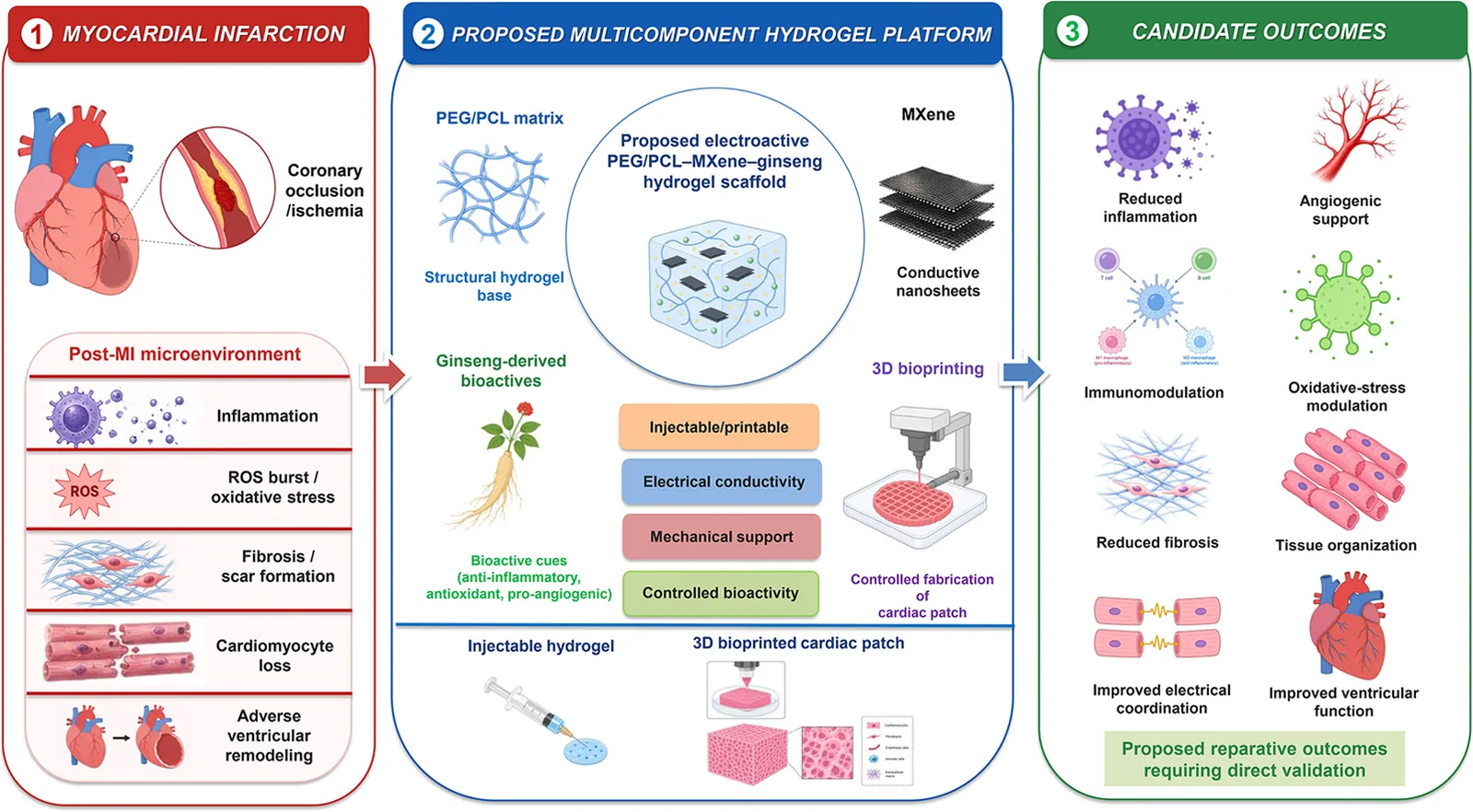

## 1 Introduction

Cardiovascular diseases (CVDs) remain the leading cause of death; myocardial infarction (MI) drives heart failure and disability [1]. Limited adult cardiomyocyte renewal after MI favors collagen-rich scar formation [2]. Repair involves inflammatory, proliferative, and maturation phases with nuclear factor kappa B (NF-κB) and mitogen-activated protein kinase (MAPK) signaling, matrix metalloproteinase (MMP) activity, extracellular matrix (ECM) turnover, fibroblast activation, angiogenesis, and scar remodeling [2]. Despite advances in acute management and secondary prevention, current therapies do not regenerate myocardium with native-like mechanical, vascular, and electrical integration [3]. MI care prioritizes timely reperfusion, hemodynamic stabilization, and evidence-based pharmacotherapy; biomaterial interventions should complement established care within clinically defined treatment windows [4]. Cardiac tissue engineering explores cell-based and cell-free approaches using injectable hydrogels, epicardial patches, and localized delivery systems [3]. Injectable hydrogels enable conformal delivery; epicardial patches provide architectural control and mechanical reinforcement [5]. Translation remains limited by poor retention, incomplete vascularization, immune activation, mechanical mismatch, uncontrolled degradation, inadequate electromechanical coupling, and uncertain durability [3].

Electroactive hydrogels aim to mitigate electrical discontinuity using graphene derivatives, carbon nanotubes (CNTs), metallic nanostructures, conductive polymers such as polyaniline (PANI), polypyrrole (PPy), and poly(3,4-ethylenedioxythiophene):poly(styrene sulfonate) (PEDOT:PSS), or MXenes [6]. MXenes are aqueous-processable, conductive two-dimensional materials [7], but oxidative instability, dose- and formulation-dependent cytotoxicity, uncertain degradation-product fate and biodistribution, and limited long-term safety evidence constrain cardiac use [8,9]. Ginseng-derived bioactives show antioxidant, anti-inflammatory, anti-apoptotic, and anti-fibrotic activity in cardiovascular models [10], although scaffold delivery requires standardized composition, dosing, release, stability, and interaction testing. Three-dimensional (3D) bioprinting provides architectural and spatial control [11], but printability alone cannot establish vascular integration, electromechanical coupling, or therapeutic efficacy. This review compares hydrogel formats, conductive materials, poly(ethylene glycol) (PEG)/poly(ε-caprolactone) (PCL) scaffolds, MXenes, ginseng-derived bioactives, and biofabrication across performance, evidence maturity, biosafety, manufacturing, quality control, and regulatory translation. The integrated PEG/PCL/MXene/ginseng platform remains a preclinical design concept, not yet a validated therapy.

## 2 Myocardial infarction pathophysiology as a biomaterial design framework

Acute ischemic injury during MI causes irreversible cardiomyocyte loss, metabolic dysfunction, and disrupted excitation-contraction coupling (Fig. 1A) [12]. Ischemia suppresses oxidative phosphorylation and adenosine triphosphate (ATP) production, increases anaerobic glycolysis and lactate accumulation, and disturbs mitochondrial redox and ionic homeostasis [12]. Dysregulated calcium transfer between the sarcoplasmic reticulum (SR) and mitochondria, mitochondrial Ca²⁺ overload, and regional changes in sarco/endoplasmic reticulum Ca²⁺-ATPase 2a (SERCA2a) and Na⁺/Ca²⁺ exchanger (NCX) expression impair Ca²⁺ cycling and contraction [13,14]. Limited cardiomyocyte renewal favors scar formation and left ventricular (LV) dysfunction [2,15]. Post-MI repair spans overlapping inflammatory, proliferative, and maturation phases (Fig. 1B) [12]. Damage-associated molecular patterns recruit neutrophils and monocyte-derived macrophages to clear necrotic tissue; fibroblasts and endothelial cells support granulation tissue, neovascularization, and ECM deposition [12]. Macrophage phenotypes vary across time and tissue regions and cannot be reduced to a binary M1/M2 classification [16]. These responses stabilize the infarct, but unresolved inflammation or excessive matrix accumulation promotes fibrosis and adverse remodeling [2]. During maturation, collagen organization and crosslinking strengthen the scar while reducing compliance, whereas repolarization heterogeneity in scar and border-zone tissue can create substrates for reentrant ventricular tachycardia [17,18]. This sequence defines phase-specific biomaterial requirements. Early materials should avoid amplifying injury without suppressing debris clearance; later performance depends on mechanical stabilization, vascular integration, matrix organization, controlled degradation, and electrophysiological compatibility [19]. Experimental measurements and finite-element modeling showed that hydrogel injection altered myocardial mechanics and reduced modeled fiber stress, with injectate stiffness, volume, and anisotropy influencing unloading [20]. This supports a mechanical rationale without demonstrating regeneration or durable functional benefit. Conductivity must be assessed alongside filler dose, dispersion, spatial uniformity, anisotropy, degradation, tissue coupling, and rhythm safety [6].

**Fig. 1:**
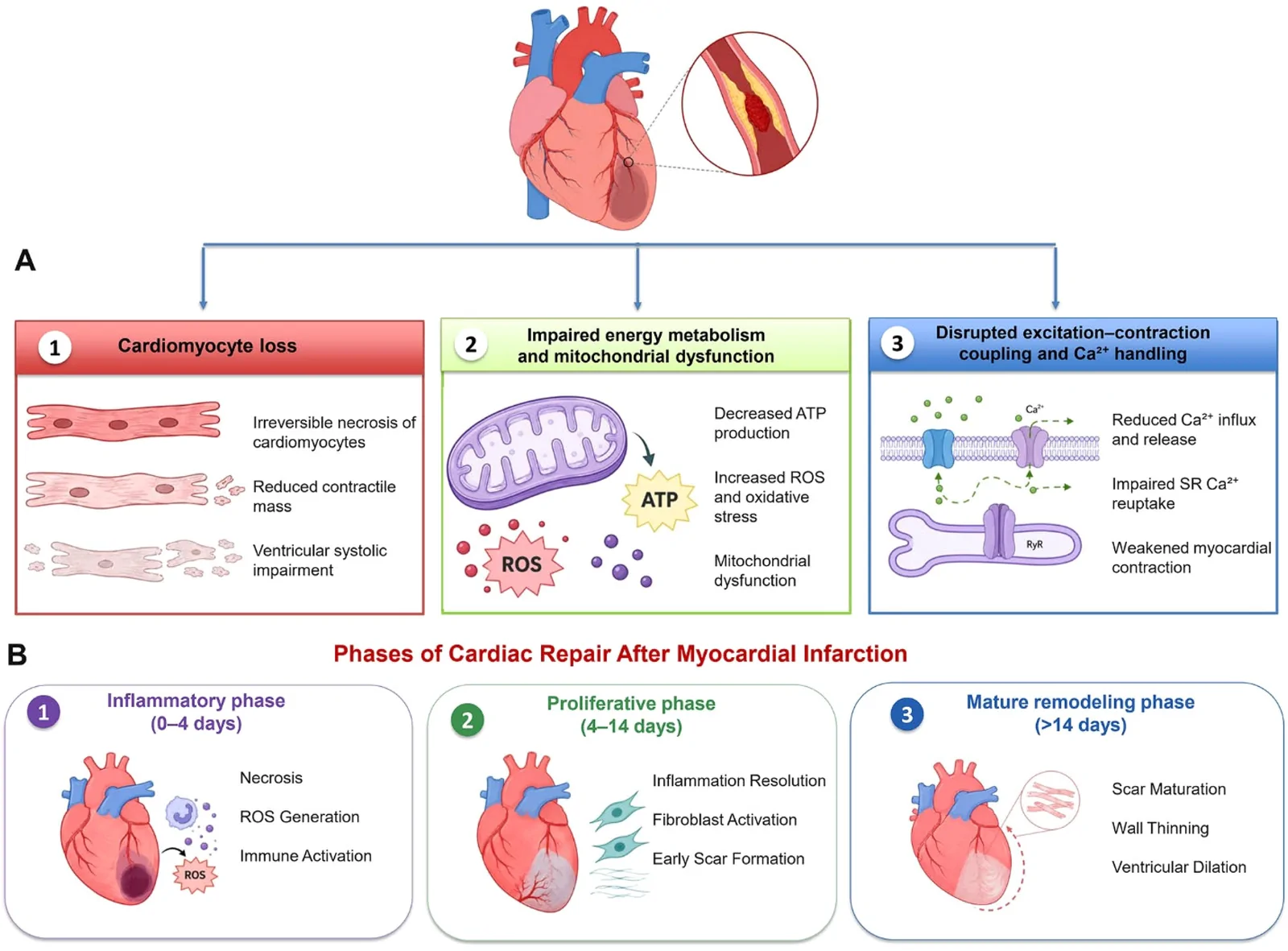
Myocardial infarction pathophysiology as a biomaterial design framework. (A) Cardiomyocyte loss, impaired energy metabolism, mitochondrial dysfunction, and abnormal Ca²⁺ handling. (B) Approximate inflammatory (0–4 days), proliferative (4–14 days), and mature remodeling (>14 days) phases; timing varies by species, infarct model, and reperfusion status. These processes motivate phase-specific mechanical, immunomodulatory, vascular, degradative, and electrical design. Created by the authors based on Yi et al. [19] and evidence [12,13,16]. **Abbreviations**: ATP, adenosine triphosphate; ROS, reactive oxygen species; RyR, ryanodine receptor; SR, sarcoplasmic reticulum.

## 3 Hydrogel platforms for myocardial repair

Hydrogels can mimic selected ECM features, retain therapeutic cargo, and provide temporary mechanical support after MI [21]. Evaluation should therefore extend beyond cytocompatibility to delivery, retention, adhesion, degradation, vascular integration, and electromechanical compatibility [5].

### 3.1 Hydrogel design, translational evidence, and benchmarks

Hydrogel behavior depends on polymer origin, architecture, charge, crosslinking, and responsiveness [21]. Physical networks can improve injectability and self-healing but may wash out, whereas covalent networks offer greater stability and degradation control but require assessment of initiators and residual reagents [19,21]. Natural hydrogels provide cell-interactive motifs but may vary between batches, degrade rapidly, or present mechanical and xenogeneic limitations [5]. Synthetic PEG/PCL matrices improve reproducibility but often require adhesive or bioactive cues; hybrid systems may balance these properties at the cost of greater analytical, manufacturing, and regulatory complexity. Most platforms remain preclinical, with large-animal and human studies providing route- and product-specific benchmarks [5]. Large-animal studies reported angiogenic and functional benefits from a bFGF-releasing gelatin sheet in canine chronic MI and catheter-delivery feasibility for myocardial ECM hydrogel in porcine MI, although long-term durability and translation to clinical benefit remain unresolved [22,23]. Algisyl-LVR improved exercise capacity and symptoms in 78 patients with advanced heart failure, although it represents surgical ventricular augmentation rather than myocardial regeneration and did not establish mortality benefit [24]. Table 1 summarizes representative platforms; differences in species, infarction models, controls, endpoints, and follow-up periods preclude direct ranking or extrapolation to cell-laden, electroactive, MXene-containing, or ginseng-loaded systems. Functional outcomes should be interpreted alongside assessments of retention, degradation, vascularization, fibrosis, immune response, arrhythmia, thrombosis, and durability [5].

**Table 1:** Representative hydrogel and scaffold-based platforms evaluated for myocardial infarction repair: composition, delivery, evidence, and limitations.

Platform	Composition/cargo	Model, delivery, timing/follow-up	Principal findings and evidence limitations	Ref.
Alginate + ASCs	In situ-forming alginate + ASCs	Rat acute MI; immediate intramyocardial injection; 28 days	ASCs improved ejection fraction; alginate added no retention/functional benefit.	[25]
Collagen matrix	Acellular injectable collagen	Mouse MI; injected at 3 h, 1 or 2 weeks; up to 3 months	Earlier injection reduced scar expansion, fibrosis, apoptosis, inflammation; increased vascular density.	[26]
Fibrin patch + BMSCs	Fibrin patch + BMSCs	Immunodeficient rat chronic MI; epicardial; 21 days	Function/angiogenesis improved; no myocardial BMSC migration; patch BMSCs in 10 % of hearts at day 21.	[27]
Gelatin sheet + bFGF	Biodegradable gelatin sheet; sustained bFGF release	Canine chronic MI; epicardial at 4 weeks post-MI; 6-week assessment	Increased capillary density/LV fractional shortening; minimally invasive feasibility/durability uncertain.	[22]
Decellularized myocardial ECM hydrogel	Acellular porcine myocardial ECM	Porcine MI; percutaneous transendocardial injection 2 weeks post-MI; 3 months	Feasible catheter delivery; improved ventricular endpoints; long-term safety/efficacy unresolved.	[23]
HA-gelatin + CDCs	In situ-polymerizable HA-gelatin + human CDCs	Mouse MI; border-zone intramyocardial injection; 3 weeks	Improved engraftment; reduced remodeling/functional decline; remuscularization unproven.	[28]
Keratin hydrogel	Acellular human hair-derived keratin	Rat acute MI; intramyocardial injection; 8 weeks	Increased angiogenesis without increased inflammation; limited remodeling, preserved function; safety/standardization unresolved.	[29]
Matrigel, fibrin, and collagen I	Three acellular natural injectable matrices	Rat ischemia–reperfusion; intramyocardial injection; 5 weeks	All similarly increased capillary formation; collagen increased myofibroblast infiltration.	[30]
Matrigel + survival cocktail + hiPSC-CMs	Matrigel + pro-survival cocktail + hiPSC-CMs	Immunodeficient rat acute MI; transplantation 1 week post-MI; 4 weeks	Matrigel/cocktail yielded largest, most mature grafts; no contractile gain; ectopic cartilage in some grafts; Matrigel limits translation.	[31]
Sericin hydrogel	Acellular crosslinked silk sericin	Mouse acute MI; immediate intramyocardial injection; 6 weeks	Improved function/structure/neovascularization; reduced scar/inflammation/apoptosis; short-term mouse evidence.	[32]
Chitosan-HA/silk fibroin patch	Acellular natural-composite patch	Rat cardiac cryoinjury; epicardial; 8 weeks	Reduced dilation; increased wall thickness, fractional shortening, neovascularization; cryoinjury/surgery limit comparison.	[33]
Silk fibroin-HA patch + BMSCs	Silk fibroin-HA patch ± BMSCs	Rat cardiac cryoinjury; epicardial; 8 weeks	Acellular patch improved remodeling; BMSCs increased wall thickness/survival/angiogenesis, reduced apoptosis; long-term fate/durability unresolved.	[34]
α-CD/MPEG–PCL–MPEG hydrogel	Acellular supramolecular α-CD/MPEG–PCL–MPEG hydrogel	Rabbit MI; intramyocardial injection 1 week post-MI; 28 days	Reduced scar expansion, wall thinning, dilation; increased ejection fraction; microvessel density unchanged.	[35]
α-CD/MPEG–PCL–MPEG + BMSCs	Supramolecular PEG/PCL + BMSCs	Rabbit acute MI; injection 1 week post-MI; 4 weeks	Improved function/remodeling, retention, vessel density; no significant hydrogel-only benefit.	[36]
FA-peptide hydrogel + iPS cells	FA-modified supramolecular peptide + iPS cells	Mouse acute MI; intramyocardial injection; 4 weeks	Improved cell retention/survival, function, remodeling, revascularization; long-term graft behavior/tumorigenicity unassessed.	[37]
Protease-responsive HA + siMMP-2	Hydrazide-/aldehyde-modified HA + siMMP-2	Rat MI; intramyocardial injection; 4 weeks	Increased infarct-zone thickness, ejection fraction, stroke volume, cardiac output; long-term off-target/degradation safety unresolved.	[38]
OPF/graphene oxide hydrogel	Acellular conductive OPF/graphene oxide	Rat chronic MI; injection 4 weeks post-MI; 4-week assessment	Improved ejection fraction/fractional shortening/electrical coupling; reduced scar; chronic electrophysiology/material fate unreported.	[39]
FPDA/PEDOT:PSS + α-tocopherol	F127-diacrylate/PEDOT:PSS + α-tocopherol	Rabbit MI; epicardial after LAD ligation; 4 weeks	Improved ejection fraction/fractional shortening; reduced infarct burden; modulated oxidative stress; epicardial-only evidence.	[40]
Gel@CK electroactive hydrogel	Natural-polysaccharide + carboxylated CNT/KRX complexes	Rat MI (LAD ligation); local injection day 0; through day 28	In vitro: ROS scavenging/pro-angiogenesis; rats: increased angiogenesis, shorter QRS, improved function, reduced oxidative stress/infarct size; chronic rhythm safety/material fate unassessed.	[41]
E-MXene injectable hydrogel	Temperature-sensitive decellularized ECM + Ti₃C₂Tₓ nanosheets	Mouse MI; early infarct injection; 28 days	Mice: reduced ROS/apoptosis, improved structure/function/electrical transmission; hiPSC-CMs in vitro: enhanced Ca²⁺ synchrony/coupling; chronic rhythm safety/biodistribution/fate unreported.	[42]
MXene/silk fibroin-HA hydrogel	Covalently crosslinked silk fibroin-HA + Ti₃C₂Tₓ	Rat MI; four peri-infarct injections; 4 weeks	Conductivity reached 0.32 S/m; improved electrical transmission/LV recovery; long-term durability/MXene fate unassessed.	[43]
Collagen/elastin/PCL patch ± c-kit⁺ cells	Electrospun collagen/elastin/PCL ± bone marrow c-kit⁺ cells	Mouse MI; epicardial patch; 4 weeks	Improved function; reduced infarct size/LV remodeling; added cell-seeding benefit; long-term fate/arrhythmia unassessed.	[44]
Bio-inert PEG hydrogel	Acellular nondegradable PEG; passive support	Rat MI; injection 1 week post-MI; assessed 49 ± 4 days post-MI	Increased infarct-wall thickness without preserving function or preventing adverse remodeling.	[45]
Col-SH/OAC–PEG–OAC ± BMSCs	Thiolated collagen/OAC–PEG–OAC ± BMSCs	Rat MI; intramyocardial injection; 28 days	Reduced scar; increased wall thickness/function; BMSCs enhanced benefit/persistence; short-term rat evidence.	[46]
PEG-fibrinogen + VEGF-A	PEG-fibrinogen; sustained VEGF-A release	Rat MI; intramyocardial injection; 4 weeks	Enhanced vascular recruitment/arteriogenesis, infarct stabilization, cardiac function; dose/long-term vascular safety unresolved.	[47]
PEG-HA + HWJMSCs/IGF-1	PEG-HA + HWJMSCs ± IGF-1	Rabbit acute MI; intramyocardial injection; 8 weeks	Improved ejection fraction/vascular density; IGF-1 strongest response, reduced inflammation; long-term cell fate/durability unresolved.	[48]
PGCL scaffold + BMMNCs	PGCL scaffold + BMMNCs	Rat MI; epicardial implantation; 4 weeks	Limited LV dilation; preserved systolic function; BMMNCs promoted migration/differentiation, neovascularization; surgery/short follow-up/cell fate limit interpretation.	[49]
pNVMT/TEMPO hydrogel	Thermoresponsive pNVMT; TEMPO radical-scavenging motif	Rat ischemia–reperfusion; injection after 30 min ischemia, followed by 4 h reperfusion	Scavenged ROS; reduced acute reperfusion injury; preserved LV geometry; chronic remodeling efficacy unproven.	[50]
Omentum-supported MSC/PLGA patch	Vascularized omental flap + MSC-seeded PLGA scaffold	Rat chronic MI; epicardial at 8 weeks post-MI; 4-week assessment	Improved MSC survival/remodeling/function; omentum alone ineffective; PLGA-specific effects not isolated.	[51]

**Abbreviations:** α-CD, α-cyclodextrin; ASCs, adipose-derived stromal cells; bFGF, basic fibroblast growth factor; BMMNCs, bone marrow-derived mononuclear cells; BMSCs, bone marrow-derived mesenchymal stem/stromal cells; CDCs, cardiosphere-derived cells; CNT, carbon nanotube; Col-SH, thiolated collagen; ECM, extracellular matrix; E-MXene, temperature-sensitive decellularized ECM/Ti₃C₂Tₓ hydrogel; FA, folic acid; FPDA, Pluronic F127-diacrylate/PEDOT:PSS/polydopamine/α-tocopherol hydrogel; Gel@CK, CNT/KRX-loaded polysaccharide hydrogel; HA, hyaluronic acid; hiPSC-CMs, human induced pluripotent stem cell-derived cardiomyocytes; HWJMSCs, human Wharton’s jelly mesenchymal stem/stromal cells; IGF-1, insulin-like growth factor-1; iPS, induced pluripotent stem cells; KRX, pro-angiogenic peptide; LAD, left anterior descending coronary artery; LV, left ventricle; MI, myocardial infarction; MPEG, methoxy poly(ethylene glycol); MSC, mesenchymal stem/stromal cell; OAC, oligo(acryloyl carbonate); OPF, oligo(poly(ethylene glycol) fumarate); PCL, poly(ε-caprolactone); PEDOT:PSS, poly(3,4-ethylenedioxythiophene):poly(styrene sulfonate); PEG, poly(ethylene glycol); PGCL, poly(glycolide-co-caprolactone); PLGA, poly(lactic-co-glycolic acid); pNVMT, poly(N-isopropylacrylamide-co-N-vinylpyrrolidone-co-methacrylate-polylactide-co-methacrylate-TEMPO); QRS, ventricular depolarization complex; ROS, reactive oxygen species; siMMP-2, small interfering RNA against matrix metalloproteinase-2; TEMPO, 2,2,6,6-tetramethylpiperidine-1-oxyl; Ti₃C₂Tₓ, titanium carbide MXene; VEGF-A, vascular endothelial growth factor-A.

Observed benefits varied by formulation and cargo, but multicomponent designs complicate attribution. Functional, vascular, or structural gains do not establish mature, electrically integrated remuscularization. In a fibrin patch, BMSCs remained confined to the patch, consistent with a paracrine reservoir mechanism [27]. Collagen increased myofibroblast infiltration despite comparable angiogenic responses across tested matrices, indicating that matrix-driven remodeling was not uniformly beneficial [30]. MXene evidence remains short-term and rodent-based, without resolving chronic biodistribution, degradation-product fate, thrombogenicity, immunogenicity, or arrhythmogenic risk [42,43]. Matrigel supports cardiac-cell engraftment [31] but is tumor-derived, xenogeneic, and batch-variable [52]. Defined PEG/PCL hybrids offer tighter control of adhesion, stiffness, degradation, loading, and electrical functionality, yet require potency assays, impurity limits, sterilization validation, and batch-release criteria. Mechanical and electrical targets are context-dependent. Human LV stiffness ranges from approximately 10–20 kPa at early diastole to 200–500 kPa at end diastole [53], whereas myocardial conductivity is approximately 5 × 10⁻⁵ S/cm transversely and 1.6 × 10⁻³ S/cm longitudinally [54]. Because values vary with loading, disease, orientation, hydration, and method, bulk measurements should be paired with deformation testing, conduction mapping, calcium-wave analysis, connexin distribution, and rhythm assessment.

### 3.2 Delivery formats and functional hydrogel strategies

Post-MI hydrogels are delivered mainly by intramyocardial or transendocardial injection or as epicardial patches [21]. Delivery choice should be guided by MI stage, therapeutic objectives, and procedural burden. In a mouse MI model, collagen matrix delivery at 3 h produced greater long-term benefit than delivery at 1 or 2 weeks, although this result does not define a clinical treatment window [26]. The first-in-human VentriGel study enrolled patients with LV dysfunction 60 days to 3 years after MI, demonstrating procedural feasibility and preliminary safety without establishing optimal timing or efficacy [55]. In the acute setting, any biomaterial intervention must be compatible with time-sensitive reperfusion and hemodynamic stabilization and should not delay established treatment [4]. During evolving remodeling, injectable depots may deliver localized bioactive or immunomodulatory cues; epicardial patches may provide mechanical reinforcement, anisotropic organization, or sustained support when surgical access is clinically justified. In established chronic scar, both approaches face mature fibrosis, reduced tissue plasticity, and challenges in durable host-material integration. Injectables offer conformal, potentially catheter-compatible delivery but risk washout and heterogeneous distribution; epicardial patches require surgical access, secure fixation, and cyclic fatigue resistance [21,56]. In rats, injected and patch formats of the same mitoquinone mesylate (MitoQ)/hydrogel system were both active, with greater benefit after injection under that protocol [57]; this does not establish route superiority across disease stages or formulations. Functional systems may incorporate conductive materials, antioxidants, immunomodulatory or angiogenic agents, nucleic acids, extracellular vesicles, or cells [19]. Conductive formulations also require evaluation of host-tissue coupling and chronic rhythm safety [6]. Adhesive, self-healing, and stimuli-responsive designs may improve retention or control release, but they also increase demands related to storage, sterilization, manufacturing, quality control, and regulatory assessment [19,56]. Route and formulation should be co-developed from the outset to ensure controlled delivery, target-site retention, material performance, and safety under the intended administration mode.

## 4 Biofabrication and 3D/4D bioprinting of cardiac constructs

Biofabrication and 3D bioprinting enable spatial control of biomaterials, cells, and biological cues [58]. Engineered heart tissue (EHT) is established for disease modeling and drug testing and is increasingly investigated for myocardial repair [11]. Geometry alone does not reproduce myocardium; maturation also requires ECM signaling, perfusion, anisotropy, electrical coupling, biochemical regulation, and mechanical loading [58]. Heart-derived decellularized ECM (hdECM) bioinks retain tissue-specific cues and have supported vascularization and repair in rodent ischemic-heart models, but donor and processing variability, residual material, batch-dependent rheology, sterility, and regulatory characterization remain unresolved [59]. Available approaches include inkjet, extrusion-based, laser-assisted, light-based, and scaffold-free bioprinting [11]. Cardiac bioinks must balance printability with cytocompatibility, gelation, compliance, degradation, nutrient transport, and electrical and contractile maturation [58]. Patient-specific induced pluripotent stem cell (iPSC)- and ECM-derived bioinks enabled thick vascularized patches and heart-like constructs (Fig. 2) [60], but mature pump-supporting constructs with clinically relevant force generation, electrophysiological stability, handling, and durable integration remain unachieved.

**Fig. 2:**
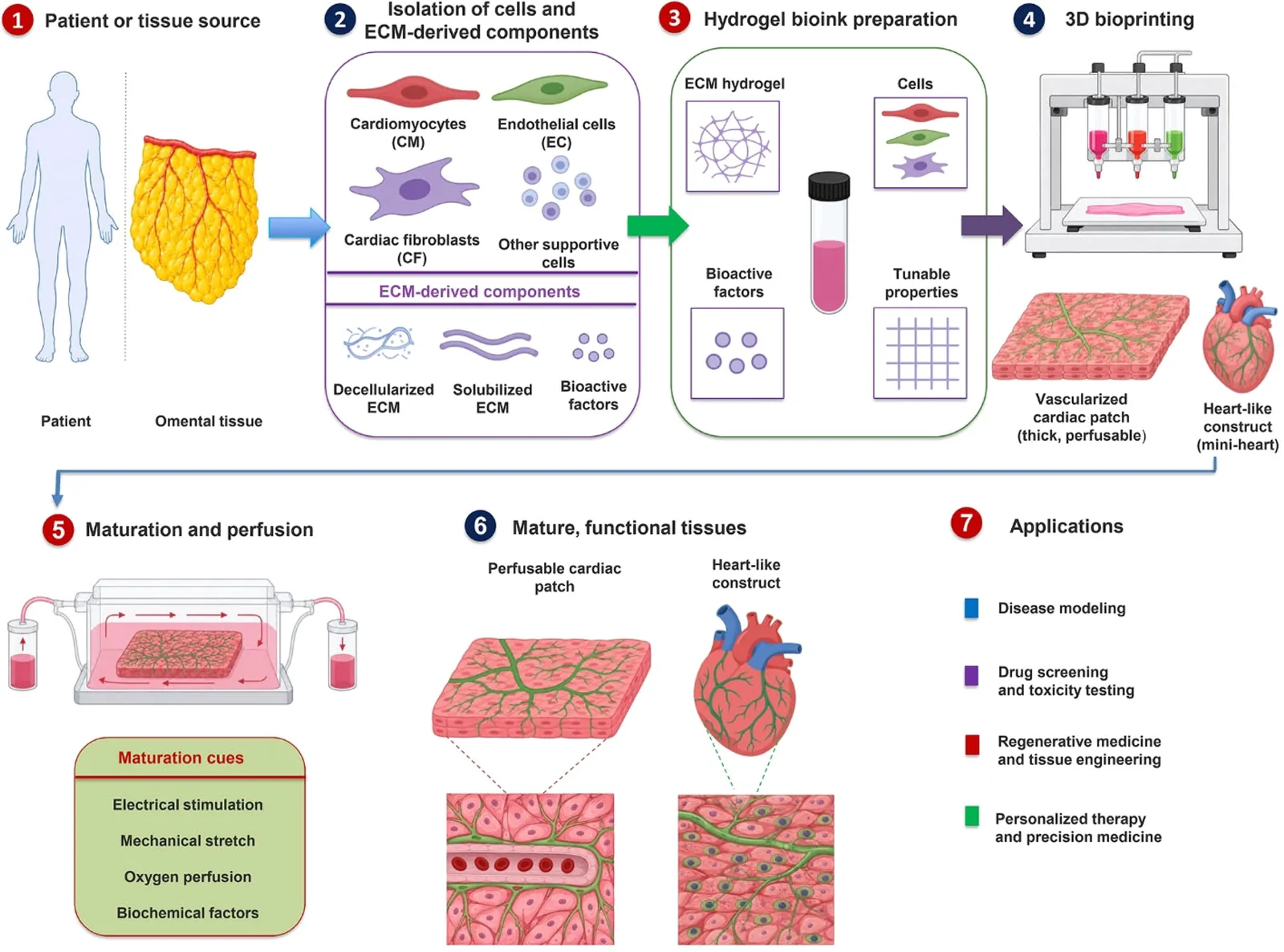
Patient-specific 3D bioprinting workflow for vascularized cardiac patches and heart-like constructs. Patient-derived cells and ECM components are formulated as bioinks, printed with defined tissue and vascular architecture, and matured under perfusion and electrical, mechanical, oxygenation, and biochemical cues. Created by the authors and conceptually informed by Noor et al. [60]. **Abbreviations**: 3D, three-dimensional; ECM, extracellular matrix.

Mesenchymal stem/stromal cell (MSC)/graphene oxide constructs and cardiomyocyte-laden PEDOT:PSS/gelatin methacryloyl (GelMA) granular hydrogels improved small-animal MI outcomes [61,62], while a structural-color hydrogel incorporating polydopamine-modified MXene combined sensing and repair [63]. However, these studies do not isolate component effects or establish long-term rhythm safety, material fate, sensor-calibration stability, or large-animal and manufacturing feasibility. Engineered heart muscle (EHM) allografts provide a more advanced translational benchmark than most acellular or bioprinted hydrogel platforms, but are not equivalent to bioprinted hydrogel patches [64]. In rhesus macaques, iPSC-derived grafts vascularized and supported cardiac function without observed arrhythmia or tumor formation [64]. In the open-label phase 1–2 BioVAT-HF study, 20 patients received allografts under immunosuppression. At 3 months, KCCQ-OSS increased by 6.7 points (P = 0.06); all patients had at least one adverse event, and three died during the study. The interim nonrandomized findings do not establish efficacy [65]. Four-dimensional (4D) biofabrication introduces programmed post-fabrication changes. A GelMA/poly(ethylene glycol) diacrylate (PEGDA) patch containing human iPSC-derived cardiomyocytes, endothelial cells, and MSCs adapted to the epicardium and was evaluated for 4 months in chronic mouse MI [66]. This supports mechanical adaptability, but perfusion, immune compatibility, tumorigenicity, arrhythmogenicity, storage, and Good Manufacturing Practice production remain unresolved. Table 2 summarizes representative cardiac patch and biofabrication platforms, their evidence levels, principal outcomes, and translational limitations.

**Table 2:** Representative cardiac patch, biofabrication, and translational benchmark systems for myocardial repair.

Patch category	Patch platform / construct	Therapeutic cargo or cellular component	Model, delivery route, and evidence scale	Principal reported outcomes and translational relevance	Ref.
Cellular EHT	Neonatal-rat-cell cardiac tissue patch	Neonatal rat ventricular cells	Rat; epicardial implantation; engraftment study, not MI efficacy	Structural/electrical properties maintained; host vascularization; no detected host–graft electrical coupling; MI efficacy untested.	[67]
Cellular / 3D-bioprinted	Biomaterial-free hiPSC-derived patch	hiPSC-CMs, fibroblasts, endothelial cells	Rat MI; epicardial implantation; 4 weeks	Engraftment, vascularization, reduced scar; echocardiographic benefit only a trend; long-term safety unassessed.	[68]
Cellular EHT	Fibrin-based EHT patch	Up to 20 million hPSC-CMs	Rabbit permanent MI; epicardial graft; 4 weeks; mid-sized animal	LV function improved; 35 % scar reduction; vascularized graft, with no detected host–graft electrical coupling or proarrhythmia.	[69]
Cellular sheet / elastic	ADSC sheet + biodegradable elastic patch	ADSCs	Rat MI; epicardial placement	Improved retention, neovascularization/function; cell-sheet versus elastic-support effects partly separable.	[70]
Cellular / conductive injectable	Intrapericardial mechanical–electrical hydrogel patch	Therapeutic cells in conductive hydrogel	Rat MI; intrapericardial delivery	Reduced fibrosis/remodeling; improved neovascularization, electrical propagation/function; cell/hydrogel effects not isolated.	[71]
Cellular / ECM-based	hdECM/SDF-eMSC patch plus intramyocardial hiPSC-EC delivery	SDF-eMSCs and hiPSC-ECs	Rat MI; epicardial patch plus intramyocardial cells; 8 weeks	Combination improved hiPSC-EC survival, neovascularization/function versus hiPSC-ECs alone; route effects not isolated.	[72]
Cellular / vascularized	Stromal-cell patch with biomimetic microvessels	Cardiac stromal cells and engineered microvessels	Rat and pig MI; preclinical, including large-animal feasibility	Better function, less scarring/inflammation, more neovascularization in rats/pigs; no perfused human graft microvessels detected in pigs.	[73]
Cellular / 4D adaptive	Physiologically adaptable GelMA/PEGDA patch	hiPSC-CMs, endothelial cells, MSCs	Mouse chronic MI; epicardial patch; 4 months	In vitro: maturation/vascularization; in vivo: engraftment/vascular supply without significant functional gain; large-animal/manufacturing validation lacking.	[66]
Cellular EHM allograft / benchmark	Engineered heart muscle allografts and BioVAT	Allogeneic iPSC-derived cardiomyocytes and stromal cells	Rhesus macaques; humans: phase 1–2 (n = 20), epicardial grafts under immunosuppression.	Macaques: 40–200 million-cell grafts persisted up to 6 months; no arrhythmia/tumor observed. Humans: 12/16 high-dose patients completed 3-month follow-up; wall +4.5 mm, LVEF +3.9 percentage points; adverse events in all 20; efficacy unproven.	[64,65]
Acellular adhesive	Viscoelastic adhesive epicardial patch	None	Rat acute/subacute MI; epicardial application	Reduced remodeling/improved function; adhesion reproducibility, mechanical durability, atraumatic placement unvalidated.	[74]
Acellular ECM	Decellularized porcine myocardial slice patch	None	Rat acute MI; epicardial implantation	Improved function/repair; xenogeneic sourcing, variable decellularization, residuals/batch release unresolved.	[75]
Acellular conductive	Anisotropic rGO/silk fibroin matrix	None	Rat MI; epicardial patch	Improved pumping, cardiomyocyte survival, ventricular-fibrillation resistance, capillary formation; chronic rGO fate unknown.	[76]
Acellular conductive / adhesive	Naturally derived conductive adhesive patch	GelMA and bio-ionic-liquid conductive adhesive	In vitro cardiac cells, ex vivo myocardium; mouse MI, epicardial patch; 3 weeks	Mouse MI: adhesion, less histological wall thinning at 3 weeks; conductivity demonstrated; functional efficacy unassessed.	[77]
Acellular drug-delivery	PLGA/gelatin multilayer patch	Adenosine	Pig ischemia–reperfusion (n = 4); epicardial patch; 24 h or 3 months (n = 1)	Local adenosine release/pro-survival signaling; feasibility only, efficacy unproven.	[78]
Acellular nitric oxide-releasing	Nitrate-functionalized biodegradable patch	Local nitric oxide delivery	Rat MI with porcine confirmation; includes large-animal evidence	Less injury/remodeling, improved function; dose, release, stability and systemic hemodynamic safety unvalidated.	[79]
Cellular / bioactive-factor	Human cardiac muscle patch	hiPSC-CMs; CHIR99021/FGF1-loaded nanoparticles	Mouse MI; epicardial patch; 4 weeks; n = 10/group	In vitro: 4–6-fold higher hiPSC-CM cycling; in vivo: ∼4-fold greater engraftment, larger grafts, increased angiogenesis, wall thickness/function; factor-specific effects not isolated.	[80]
Acellular bioactive-factor	PCL/collagen I heart patch	Substance P and IGF-1C peptide	Mouse MI; epicardial placement; 14 days	Improved function, neovascularization, wall thickness/remodeling; long-term cargo durability unresolved.	[81]
Acellular microneedle	GelMA hydrogel microneedle patch	Galunisertib	Rat MI; epicardial microneedle patch 10 days post-MI; 28-day assessment	Release >2 weeks; less fibrosis; patch improved function, but galunisertib added no significant LVEF/LVFS benefit over unloaded patch; chronic safety unassessed.	[82]
Cellular / conductive 3D-printed	3D-printed conductive MSC@GO patch	MSCs and graphene oxide	Mouse MI; epicardial printed patch	Conductive cell-laden patch improved repair; cell/GO/architecture effects not isolated.	[61]
Cellular / conductive 3D-printed	Granular composite myocardial patch	Cardiomyocyte microgels in PEDOT:PSS/GelMA	Rat MI; epicardial cell-laden printed patch	Cell function maintained; improved remodeling/ventricular performance; chronic PEDOT:PSS safety/graft outcomes insufficiently assessed.	[62]
Acellular MXene adhesive	Paintable adhesive MXene hydrogel patch	Ti₃C₂Tₓ MXene in gelatin/dextran-aldehyde	Rat infarcted-heart model; epicardial painting	Adhesion/support/conduction; improved function/remodeling; oxidation, biodistribution, electrophysiological safety/sterilization unresolved.	[83]
Acellular theranostic conductive	Conductive structural-color hydrogel patch	HPC cholesteric liquid crystal and polydopamine-modified MXene	Preclinical MI model; conductive sensing and repair patch	Spatiotemporal monitoring; reduced remodeling/increased vascular regeneration; calibration, storage, manufacturing, regulatory challenges remain.	[63]

**Abbreviations:** 3D, three-dimensional; 4D, four-dimensional; ADSCs, adipose-derived stem cells; BioVAT, biologic ventricular assist tissue; ECM, extracellular matrix; EHM, engineered heart muscle; EHT, engineered heart tissue; FGF1, fibroblast growth factor 1; GelMA, gelatin methacryloyl; GO, graphene oxide; HPC, hydroxypropyl cellulose; hdECM, heart-derived decellularized extracellular matrix; hiPSC-CMs, human induced pluripotent stem cell-derived cardiomyocytes; hiPSC-ECs, human induced pluripotent stem cell-derived endothelial cells; hPSC-CMs, human pluripotent stem cell-derived cardiomyocytes; IGF-1C, insulin-like growth factor-1C; LV, left ventricle; LVEF, left ventricular ejection fraction; LVFS, left ventricular fractional shortening; MI, myocardial infarction; MSCs, mesenchymal stem/stromal cells; PCL, poly(ε-caprolactone); PEDOT:PSS, poly(3,4-ethylenedioxythiophene):poly(styrene sulfonate); PEGDA, poly(ethylene glycol) diacrylate; PLGA, poly(lactic-co-glycolic acid); rGO, reduced graphene oxide; SDF-eMSCs, SDF-1α-secreting engineered mesenchymal stem cells; Ti₃C₂Tₓ, titanium carbide MXene.

Freeform reversible embedding of suspended hydrogels (FRESH) v2.0 enabled unmodified collagen features near 20 μm and neonatal-scale heart-like structures [84], a method-specific achievement rather than a universal target. For PEG/PCL/MXene/ginseng constructs, polymer composition, MXene loading and flake size, and ginseng carriers can alter rheology, nozzle stability, crosslinking, print fidelity, cell viability, conductivity, release, and maturation. Fabrication must also ensure wet adhesion, fatigue resistance, and controlled cargo release [56].

## 5 Functional material components for electroactive hydrogel scaffolds

The complete PEG/PCL/MXene/ginseng scaffold remains untested in MI models; component or partial-formulation evidence cannot establish its efficacy, additive effects, or synergy. Simultaneous changes in matrix composition, conductive additives, bioactive cargo, and fabrication can confound attribution of component effects or interactions, as illustrated by multifunctional cardiac constructs [63]. Demonstrating added value requires factorial or appropriately matched comparisons with simpler formulations, controlling dose, matrix properties, fabrication, delivery, and mechanical, electrophysiological, biological, and translational testing conditions.

### 5.1 PEG/PCL-based matrices for injectable and printable scaffolds

PEG is widely used because its hydrophilicity, low nonspecific protein adsorption, and chemical versatility permit controlled network formation, degradable crosslinks, ligand conjugation, and cargo retention [85]. Cardiac studies also expose its limitation: unmodified PEG is biologically passive. A nondegradable PEG hydrogel increased infarct-wall thickness without preventing adverse remodeling [45], whereas degradable, cell-interactive, or pro-angiogenic PEG systems produced broader effects [46,47]. PCL supports scaffold processing and reinforcement, although its hydrophobicity and slow degradation can limit hydration, cell interactions, and timely remodeling; PEG/PCL combinations partly offset these limitations [86]. Thermoresponsive PCL-PEG copolymers can remain flowable during delivery and gel near physiological temperature, although transition temperature, injectability, strength, and release depend on block length, concentration, and architecture [86]. Poly(glycerol sebacate) (PGS)/PCL/reduced graphene oxide (rGO) scaffolds showed formulation-dependent changes in fiber morphology, conductivity, degradation, and mechanics [87]. PCL-PEG-PCL (PCEC) scaffolds showed tunable porosity, cytocompatibility, degradation, dimensional accuracy, and elastic moduli of 338–705 MPa [88]. These values exceed the kilopascal-scale behavior of soft myocardium, making PCEC-rich structures more credible as reinforcing frameworks than as bulk myocardium-mimetic hydrogels [53,88]. The comparison remains qualitative because test mode and material state differ. Injectables require controlled gelation, acceptable injection force, retention, and release [23,86]; epicardial patches require adhesion, fatigue resistance, anisotropy, and timed degradation [56,74]; cell-laden prints require shear tolerance and viability [58]. MXene or ginseng-derived cargo should be retained only if it improves predefined function without destabilizing rheology, mechanics, degradation, or manufacturing reproducibility.

### 5.2 MXene-enabled electroactivity: comparative position, cardiac evidence, and safety

MXenes are two-dimensional transition-metal carbides, nitrides, and carbonitrides produced mainly by removing the A layer from MAX-phase precursors, followed by intercalation and delamination [7,89]. Their high electronic conductivity, hydrophilic surface terminations, large interfacial area, and aqueous processability distinguish them from many hydrophobic fillers [7]. Nevertheless, MXene is not a uniform biomedical material: composition, flake size, defects, terminations, residual etchants, interlayer species, oxidation state, purification, and storage history can alter dispersion, conductivity, degradation, and biological response [8,9]. Cardiac evidence spans distinct levels. A Ti₂C-containing cryogel promoted cardiomyocyte maturation and improved repair in a rodent MI model [90]. Aerosol-jet deposition of Ti₃C₂Tₓ onto PEG hydrogels generated spatially patterned conductive paths (Fig. 3) and improved cardiomyocyte alignment and functional markers, supporting PEG/MXene fabrication but not post-MI efficacy [91]. Injectable and adhesive MXene hydrogels have shown short-term functional benefit in rodents [42,83]. These studies justify continued evaluation but do not establish chronic safety, superiority, or clinical readiness. Conductive hydrogel performance depends on network type, flexibility, hydration, mechanical hysteresis, environmental stability, and sensing or signal fidelity [92]. Table 3 compares design value and translational limitations.

**Fig. 3:**
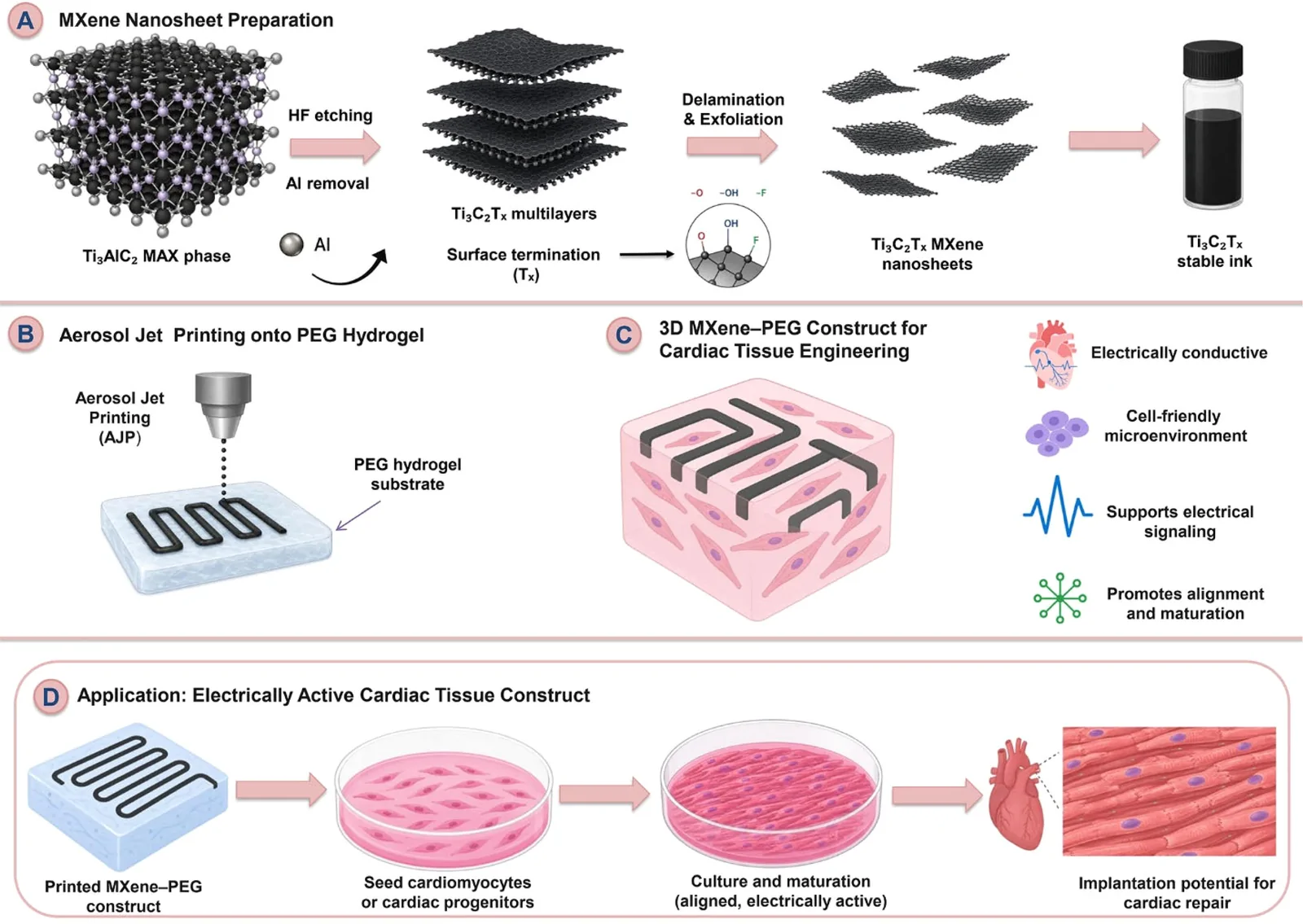
MXene-poly(ethylene glycol) (PEG) constructs for electroactive cardiac tissue engineering. Selective A-layer removal from Ti₃AlC₂, followed by intercalation and delamination, yields Ti₃C₂Tₓ nanosheets that can be aerosol-jet patterned on PEG hydrogels to create spatial conductive features intended to guide cardiomyocyte alignment and signal propagation. Created by the authors based on Basara et al. [91]. **Abbreviations:** 3D, three-dimensional; HF, hydrofluoric acid.

**Table 3:** Comparative features of conductive biomaterial classes relevant to electroactive cardiac hydrogel and patch design.

Conductive material class	Representative examples	Design value	Principal bioperformance and safety limitations	Evidence position for MI repair	Ref.
MXene-based systems	Ti₃C₂Tₓ, Ti₂C; MXene–PEG, MXene–gelatin/dextran, MXene–silk fibroin/HA hydrogels	High conductivity; hydrophilic, functionalizable surfaces; aqueous dispersion, reinforcement, patternability.	Oxidation/conductivity loss, aggregation, residual etchants, variable surface terminations; uncertain chronic degradation, biodistribution, clearance, thrombogenicity, rhythm safety.	MXene–PEG in vitro; short-term rodent MI; no chronic large-animal or clinical validation.	[8,9,43,83,90,91]
Graphene derivatives	GO, rGO, functionalized graphene composites	Tunable surface chemistry; electrical/mechanical reinforcement of fibrous or hydrogel cardiac matrices.	Size-, oxidation-, impurity-, dose-, and dispersion-dependent aggregation/biological effects; uncertain persistence/clearance.	Rodent MI proof of concept; reproducibility/material fate limit implant translation.	[6,39,76]
Carbon nanotubes	Single-/multi-walled CNTs; functionalized CNT–hydrogel composites	High aspect ratio, conductivity, reinforcement; may support cardiac/endothelial cells when dispersed.	Aggregation, persistence, dose-dependent cytotoxicity/inflammation; uncertain biodegradation/clearance.	Rodent MI proof of concept; chronic implant safety and scalable manufacturing unvalidated.	[6,41]
PPy-based systems	PPy, PPy–chitosan, PPy composites	Selected formulations support electrical signaling and synchronized contraction.	Limited biodegradability; dopant dependence, brittleness, poor solubility/dispersion, possible chronic inflammation.	Cardiac proof of concept includes rat cryoinjury (not ischemic MI); no clinical MI validation.	[93,94]
PANI-based systems	PANI, aniline oligomers, PANI composites	Redox-responsive conductivity; simple synthesis; blends with degradable matrices.	pH-dependent conductivity; poor biodegradability/solubility, brittleness, potential dopant/residual-monomer toxicity.	Established benchmark; cardiac performance/safety depend on formulation.	[93]
PEDOT/PEDOT:PSS systems	PEDOT, PEDOT:PSS, collagen–PEDOT:PSS, PEDOT:PSS/GelMA	Electrical stability, aqueous processability; compatible with soft bioelectronics/cell-laden constructs.	Not inherently degradable; possible PSS acidity/leaching, variable extensibility; sterilization/chronic response unvalidated.	Mouse MI: reduced post-infarct ventricular tachycardia; no established clinical MI product.	[62,95]
Metallic nanomaterials	Gold nanoparticles, nanorods, nanowires	High conductivity; stable, tunable interfaces for cardiac-cell organization/coupling.	Nondegradable; possible persistence/off-target accumulation, cost, concentration-dependent effects.	Gold-nanorod cardiac constructs studied in vitro; MI treatment efficacy unproven.	[6,96]
Ionic conductive hydrogels	Ion-loaded, zwitterionic, bio-ionic-liquid, ionically crosslinked hydrogels	Soft, hydrated, stretchable conduction; tissue-like interfaces during deformation.	Ion leakage, dehydration, electrochemical drift, osmotic effects; uncertain chronic cyclic stability.	Rodent MI patch benefit; adhesive-platform study lacked functional efficacy testing; chronic electrical validation limited.	[77,92,97]
Multifunctional platforms	Conductive adhesives, theranostic, peptide/CNT and sensing–delivery constructs	Combine conduction with adhesion, sensing, antioxidant/angiogenic activity or cargo release.	Component attribution difficult; calibration, sterilization, storage, manufacturing/regulatory burdens; clinical added value unproven.	Preclinical multicomponent systems; each function requires proven benefit and measurable quality attributes.	[41,63]

**Abbreviations**: CNT, carbon nanotube; GelMA, gelatin methacryloyl; GO, graphene oxide; HA, hyaluronic acid; MI, myocardial infarction; PANI, polyaniline; PEDOT, poly(3,4-ethylenedioxythiophene); PEDOT:PSS, poly(3,4-ethylenedioxythiophene):poly(styrene sulfonate); PEG, poly(ethylene glycol); PPy, polypyrrole; PSS, poly(styrene sulfonate); rGO, reduced graphene oxide; Ti₂C, titanium carbide MXene; Ti₃C₂Tₓ, surface-terminated titanium carbide MXene (Tₓ denotes surface functional groups).

Conductive polymers have a longer tissue-engineering history but remain constrained by formulation-dependent biodegradability, dopants, residual monomers, brittleness, acidity, or chronic tissue response [93]. PPy-chitosan improved synchronized contraction and impulse propagation [94], whereas collagen-PEDOT:PSS supported hiPSC-derived cardiomyocytes and reduced post-infarct ventricular tachycardia in mice [95]. No conductive class is universally optimal; performance remains formulation- and test-dependent [6]. Cell-level evaluation should pair conductivity with impedance, anisotropy, conduction velocity, calcium-wave synchronization, action-potential propagation, and connexin-43 distribution. Implant studies should additionally report host-material contact, electrophysiological mapping, programmed stimulation, telemetry, and arrhythmia burden. Patterning may reduce the need for uniform filler loading, but print fidelity does not establish conductivity retention after oxidation, sterilization, cyclic loading, or implantation, nor therapeutic benefit in infarcted myocardium [91].

A paintable gelatin/dextran-aldehyde/Ti₃C₂Tₓ patch combined adhesion, support, and conduction in rat MI [83]. An injectable ECM-derived E-MXene hydrogel reduced reactive oxygen species (ROS), improved calcium synchronization and connexin-43 expression in vitro, and improved mouse outcomes over 28 days [42]. A methacrylated silk fibroin/hyaluronic acid formulation showed concentration-dependent conductivity, swelling, degradation, and mechanics; the selected SH-M5 formulation reached approximately 0.32 S/m, showed about 3 % hemolysis in a 2-h assay, and improved 4-week rat echocardiographic indices [43]. Higher MXene loading increased aggregation, pore disruption, mass loss, and impaired mechanical recovery, showing that greater initial conductivity was not uniformly beneficial; conversely, low loading may fail to form a conductive network, whereas excess loading may also impair cytocompatibility, injectability, or printability [43]. However, cytocompatibility and baseline conductivity alone do not establish sustained electroactivity: oxidation, structural damage, and loss of hydrogel-filler contact may disrupt conductive pathways, while water, oxygen, temperature, pH, defects, flake size, surface terminations, concentration, storage conditions, and post-MI factors (including reactive oxygen species, inflammatory enzymes, proteins, macrophage activity, and repetitive mechanical strain) may alter Ti₃C₂Tₓ integrity and conductivity [9,42,43]. Accordingly, longitudinal testing should assess conductivity under oxidative and inflammatory exposure, physiological hydration, scaffold degradation, and cyclic deformation. Because heterogeneous conductive domains could create nonphysiological pathways, chronic safety should be assessed using optical or electrode mapping, programmed stimulation, and telemetry; this remains a testable risk hypothesis, not evidence of inherent proarrhythmia. Biosafety assessment should extend beyond cell viability because residual etchants, released nanosheets, and degradation products may interact with immune cells, endothelium, platelets, complement, and plasma proteins [[7], [8], [9]]. Injectable products require hemolysis, coagulation, platelet/complement activation, embolic-risk, systemic-exposure, biodistribution, and clearance testing. Epicardial products additionally require assessment of pericardial inflammation, encapsulation, adhesion durability, and particle release under cardiac motion. MXene should therefore be positioned as an emerging aqueous-processable additive, not a universally superior or clinically validated cardiac material.

### 5.3 Ginseng-derived bioactives for post-MI microenvironment modulation

Structural and electroactive functions cannot fully address post-MI oxidative stress, dysregulated inflammation, apoptosis, endothelial dysfunction, fibrosis, or adverse remodeling. Ginseng-derived bioactives are therefore candidate adjunct cargo for microenvironmental modulation, not structural or conductive components [10]. Findings from free compounds, extracts, or systemic administration cannot be directly extrapolated to scaffold delivery; selected bioactives require defined identity, purity, dose, analytical traceability, and demonstrated stability and compatibility with fabrication, sterilization, and storage. Interactions with the polymer network and conductive phase may alter compound retention, diffusion, release, bioavailability, and biological activity; therefore, antioxidant, anti-inflammatory, or cardioprotective effects observed in pharmacological models provide mechanistic support for further development, not evidence of retained efficacy, safety, or reproducibility in a multicomponent cardiac scaffold. Ginsenoside composition and abundance vary with Panax species, plant part, cultivar, and processing conditions, while analytical methods determine which compounds can be resolved and quantified [[98], [99], [100], [101]]; reproducible scaffold specifications should therefore define the chemical fingerprint, marker compounds, purity, and potency. Whole extracts may preserve broader multicomponent pharmacology, but their chemical heterogeneity complicates identity testing, potency assignment, impurity control, and batch-to-batch comparability [10,101]. By contrast, purified ginsenosides such as Rb1, Rg1, Re, Rd, and Rg2 provide a more chemically defined starting point for dose-response, stability, loading, and release studies [10]. Available cardiac evidence supports biological plausibility (Fig. 4) but remains heterogeneous in formulation and evidence maturity. Panax ginseng attenuated fibrosis and inflammation after experimental MI through sirtuin 1 (SIRT1)-associated signaling [102], although this study evaluated pharmacological activity rather than delivery from an electroactive scaffold. More directly relevant to biomaterial-based delivery, an Rb1/polydopamine nanoparticle-loaded carboxylated chitosan hydrogel modulated mitochondrial DNA (mtDNA)-stimulator of interferon genes (STING) crosstalk, macrophage phenotype, oxidative stress, fibrosis, and adverse remodeling in a preclinical MI model [103]. A more recent study used a transglutaminase-responsive injectable conductive hydrogel for sustained local delivery of ginsenoside Rg5 in a rat MI model; the formulation attenuated fibrosis, improved left ventricular electrical homogeneity, and was associated with changes in cyclic guanosine monophosphate-protein kinase G (cGMP-PKG) signaling [104]. Together, these studies provide proof of principle that defined ginsenosides can be incorporated into local hydrogel-based cardiac delivery systems under preclinical conditions. Because the studies differ in ginsenoside identity, carrier composition, conductive architecture, delivery design, and experimental conditions, they do not establish the optimal bioactive, dose, carrier, release profile, or treatment window for a PEG/PCL/MXene scaffold; release kinetics should instead be tailored to the temporal biology of MI [12]. In the final scaffold formulation, loading efficiency, free and bound fractions, release in protein-containing media, degradation-coupled release, retained potency, local tissue exposure, and systemic exposure should be quantified [105]. Stability-indicating analytical methods should distinguish parent ginsenosides from degradation products after fabrication, sterilization, storage, and release, while mechanism-linked potency assays should verify that the intended bioactivity is retained in the presence of MXene. Translation requires assessment of pharmacokinetic and pharmacodynamic interactions with standard post-MI therapies, including antiplatelet, anticoagulant, lipid-lowering, and neurohormonal drugs [106]. In a randomized controlled study in healthy participants, American ginseng reduced warfarin's anticoagulant effect [107]; although this finding cannot be extrapolated directly to other Panax species, purified ginsenosides, or local hydrogel delivery, it underscores the need to assess systemic exposure and interaction potential for the final scaffold formulation. In addition to establishing a formulation-specific dose-exposure-response relationship, the final construct must demonstrate that incorporation of the bioactive does not compromise scaffold conductivity, rheology, degradation, cytocompatibility, or manufacturing reproducibility. Direct comparison with the corresponding scaffold lacking ginseng-derived cargo is necessary to determine whether the bioactive provides measurable added value beyond the underlying scaffold. Current evidence remains preclinical and formulation-specific and does not establish large-animal feasibility, human safety, or clinical efficacy of a ginseng-loaded cardiac scaffold.

**Fig. 4:**
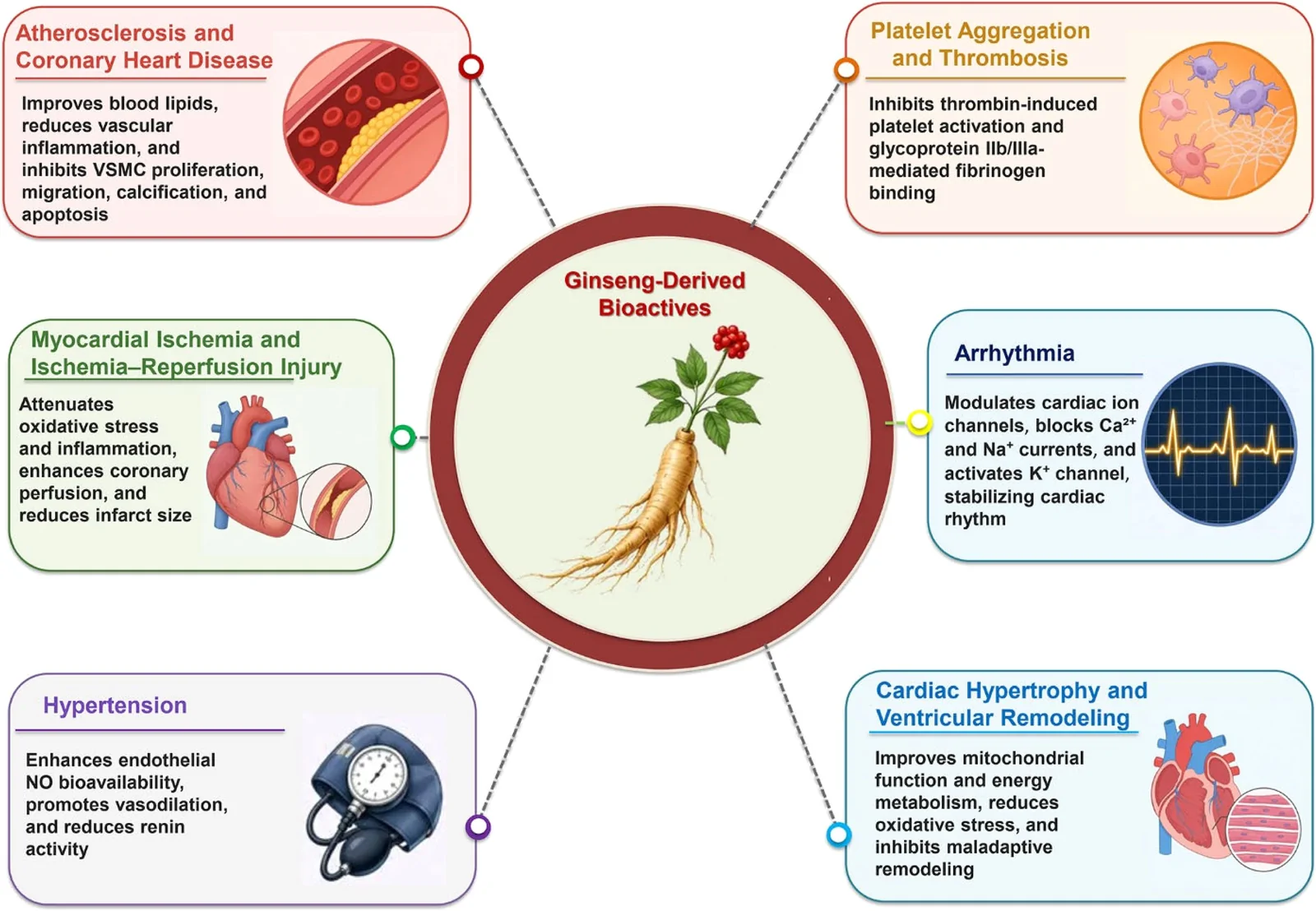
Cardiovascular pathways reported for ginseng-derived bioactives that are relevant to post-MI microenvironment modulation. Broad pharmacological evidence supports candidate effects on oxidative stress, inflammation, apoptosis, endothelial dysfunction, platelet activity, fibrosis, and remodeling [10,102]. Preclinical hydrogel-delivery studies include Rb1 and Rg5 formulations [103,104]. These findings do not validate the proposed PEG/PCL/MXene/ginseng scaffold. Created by the authors. **Abbreviations**: MI, myocardial infarction; NO, nitric oxide; VSMC, vascular smooth muscle cell.

PEG/PCL controls processing, mechanics, degradation, and release [86,88]. MXene can provide spatially controllable electroactivity but entails formulation-dependent concerns about oxidation, dosing, electrophysiological safety, and long-term material fate [8,43,91]; and ginseng-derived bioactives may modulate inflammatory, oxidative, vascular, and fibrotic pathways if identity, potency, and release are controlled [10,103]. Fig. 5 presents these complementary but non-interchangeable functions as an evidence-informed framework, distinguishing component-level support from hypothetical outcomes of the unvalidated complete scaffold.

**Fig. 5:**
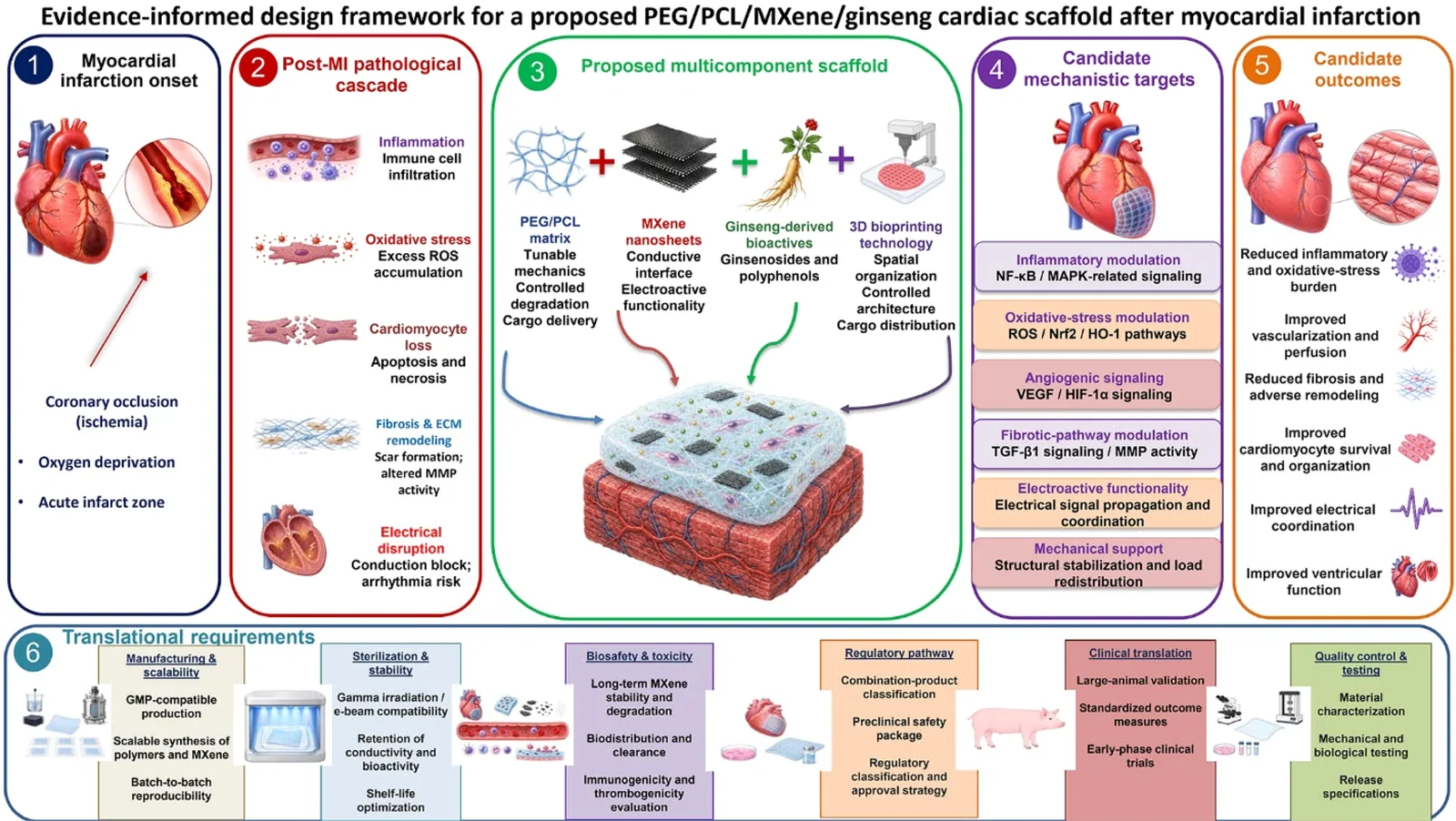
Evidence-informed design framework for a proposed PEG/PCL/MXene/ginseng scaffold after myocardial infarction (MI). The schematic links post-MI injury to a PEG/PCL matrix containing MXene nanosheets and ginseng-derived bioactives, fabricated through three-dimensional (3D) bioprinting. Candidate targets include inflammatory and oxidative pathways, angiogenesis, fibrosis, electrical coordination, and mechanical stabilization. Hypothetical outcomes include lower inflammation and oxidative stress, improved perfusion, reduced fibrosis and remodeling, enhanced cardiomyocyte survival and organization, and better electrical and ventricular function. These outcomes derive from component or partial-formulation evidence; the complete scaffold remains unvalidated. Translational requirements include scalable manufacturing, sterilization and stability testing, chronic biosafety and material-fate evaluation, regulatory classification, large-animal validation, standardized endpoints, and quality control. Created by the authors. **Abbreviations**: e-beam, electron beam; ECM, extracellular matrix; GMP, Good Manufacturing Practice; HIF-1α, hypoxia-inducible factor 1 alpha; HO-1, heme oxygenase-1; MAPK, mitogen-activated protein kinase; MI, myocardial infarction; MMP, matrix metalloproteinase; NF-κB, nuclear factor kappa B; Nrf2, nuclear factor erythroid 2-related factor 2; PEG/PCL, poly(ethylene glycol)/poly(ε-caprolactone); ROS, reactive oxygen species; TGF-β1, transforming growth factor beta 1; VEGF, vascular endothelial growth factor.

## 6 Translational readiness and regulatory considerations

Although electroactive and bioactive cardiac scaffolds vary in translational maturity, evidence largely comprises material characterization, in vitro studies, and short-term rodent findings on cell survival, angiogenesis, fibrosis, ventricular remodeling, and systolic performance; clinical benefit remains unproven [5]. Human MI is more heterogeneous, with outcomes shaped by infarct size and location, reperfusion status, ventricular function, scar maturity, comorbidities, chronic pharmacotherapy, and pre-existing arrhythmic substrate. Clinical relevance depends on material performance within the care pathway, because acute MI, evolving remodeling, and established ischemic scar differ in therapeutic priorities, acceptable procedural burden, safety requirements, comparators, and outcome measures. Translational assessment should distinguish material proof of concept, small-animal efficacy, clinically relevant large-animal delivery and safety, early human feasibility, and adequately powered evidence of clinical benefit. Supplementary Table S1 summarizes evidence stage, principal bottlenecks, and quality-control needs.

Decellularized myocardial ECM hydrogels progressed from catheter delivery in infarcted pigs to the first-in-human VentriGel study, which established transendocardial delivery feasibility and preliminary safety but not efficacy [23,55]. AUGMENT-HF reported functional benefit after surgical implantation of a permanent alginate hydrogel; however, thoracotomy and 8.6 % 30-day surgical mortality limit generalizability and position it as ventricular augmentation rather than myocardial regeneration [108]. Engineered heart muscle allografts and BioVAT-HF provide more advanced benchmarks for cell manufacturing, dose selection, immunosuppression, imaging, rhythm surveillance, and adverse-event reporting [64,65].

Before pivotal trials, specify the target product profile: material characteristics, intended use, indication, population, MI stage, treatment window, route, dose, delivery device, mechanism of action, shelf life, and clinically relevant comparator. Injectable systems require controlled gelation, acceptable catheter or needle force, homogeneous distribution, target-site retention, and low leakage or embolic risk. Epicardial patches require atraumatic fixation or reproducible wet adhesion, resistance to cyclic displacement and fatigue, conformability to the infarcted surface, and stable function on the beating heart [56], whereas printed constructs add requirements for shear tolerance, dimensional reproducibility, nutrient transport, maturation, sterility, and surgical handling [58].

Critical quality attributes should be measured in the final sterilized formulation rather than inferred from isolated ingredients. For PEG/PCL, these include polymer identity and ratio, molecular-weight distribution, crosslink density, residual reagents, rheology, swelling, modulus, fatigue behavior, degradation, and release kinetics [86]. MXene specifications should include precursor purity, etchant residues, flake-size distribution, layer number, surface terminations, oxidation state, endotoxin, dispersion, conductive uniformity, and conductivity retention after sterilization and storage [8,9]. For the final formulation, cargo loading, release, stability, and mechanism-linked potency should be verified [98,103]. Interactions among phases are also product attributes: MXene may alter crosslinking, rheology, adsorption, or release, whereas PCL-rich domains may affect hydration, degradation, and local exposure.

Multicomponent hydrogel characterization requires complementary methods to resolve interacting structural and compositional properties [109]. Inconsistent characterization and release testing limit cross-study comparisons; methods require purpose-specific validation for sensitivity, selectivity, complexity, cargo suitability, and combined-product performance [105,110]. Complementary assays should link chemical identity, cargo release, MXene dispersion, conductive uniformity, rheology, mechanics, degradation, potency, and component interactions to biological performance. Biological evaluation should use the final product or a representative test article and account for contact duration, degradation, nanoscale constituents, and possible leachables [111]. Batch-release criteria should verify product identity, sterility, endotoxin, and post-sterilization shelf life, alongside the relevant validated quality attributes under the intended manufacturing process.

Under the U.S. Food and Drug Administration framework, products containing two or more constituent parts, including drug, device, or biological components, may be regulated as combination products; the FDA center with primary jurisdiction is generally determined by the product’s primary mode of action [112]. Additively manufactured device-like components require validated design and process controls, material characterization, dimensional reproducibility, and performance testing [113]. Incorporating living cells would create a substantially different development path from an acellular PEG/PCL/MXene/ginseng patch, and each added component should provide a predefined, independently measurable contribution.

Comparative testing should include PEG/PCL alone, PEG/PCL/MXene, PEG/PCL/ginseng, the full formulation, and clinically relevant controls under matched fabrication and testing conditions. Initial studies should define dose–response relationships for MXene and the selected ginsenoside, verify retained conductivity and biological potency after sterilization and storage, and assess mechanical and electrical performance under physiological cyclic loading. In vivo evaluation should include continuous electrocardiographic telemetry, programmed stimulation, conduction mapping, hemocompatibility, thrombogenicity, immune profiling, quantitative biodistribution, and degradation-product analysis [111]. Large-animal MI models should assess clinically feasible delivery, infarct-size dependence, wall-motion effects, retention, chronic rhythm safety, and compatibility with antiplatelet, anticoagulant, lipid-lowering, and neurohormonal therapies. Advancement should follow predefined go/no-go criteria rather than isolated improvements in ejection fraction or scar size. Loss of conductivity after aging, unacceptable arrhythmia burden, uncontrolled systemic exposure, nonreproducible release, or batch-dependent mechanics should trigger program termination or reformulation despite favorable short-term functional signals.

Preclinical design should account for heterogeneity that weakens cross-study comparison by including both sexes, clinically relevant age and comorbidity where feasible, ischemia-reperfusion and permanent-ligation models, and treatment windows reflecting realistic diagnosis and intervention. Randomization, blinded outcome assessment, prespecified exclusion criteria, transparent reporting of mortality and technical failures, and power calculations based on a primary endpoint are essential to distinguish treatment effects from model variability [114].

Although left ventricular ejection fraction (LVEF) is a widely used measure of global systolic performance, its dominance as a primary endpoint in preclinical MI studies warrants caution. LVEF is volume-derived, influenced by loading conditions, ventricular geometry, and image quality, and does not directly quantify myocardial contractility [115]. In regenerative studies, the global nature of LVEF may obscure regional functional changes within the infarct and border zones; deformation imaging can detect regional or global myocardial changes not reflected by LVEF alone [116]. Endpoint selection should therefore reflect the biological question, treatment mechanism, and MI stage rather than defaulting to LVEF as the dominant efficacy measure. In acute ischemia-reperfusion cardioprotection studies, consensus recommendations identify infarct size relative to the area at risk as the principal endpoint, with ventricular function as complementary information; longer-term studies should also assess scar burden and ventricular remodeling [114]. In cardiac scaffold studies, LVEF should be interpreted alongside ventricular volumes, regional strain, infarct or scar size, wall thickness, perfusion, rhythm burden, and histological integration at time points sufficient to capture scaffold degradation and late remodeling. Independent core-laboratory analysis and multisite replication would strengthen evidence that performance is not dependent on individual operators, imaging protocols, or material batches.

Delivery should be evaluated using the intended catheter, applicator, fixation method, imaging guidance, and operator workflow rather than laboratory substitutes. Human-factors testing should assess preparation time, mixing or loading errors, premature gelation, nozzle or catheter obstruction, patch misplacement, and failure management. For nonretrievable products, uncertainty in degradation, release, or arrhythmic behavior carries greater weight than for removable devices. Clinical benefit must justify procedural burden, cell-related immunosuppression, repeated imaging, and manufacturing cost. Early trials should prioritize feasibility, dose escalation, device- or procedure-related adverse events, sustained rhythm monitoring, and predefined stopping rules before efficacy claims. Subsequent controlled studies should use population-appropriate endpoints, including functional capacity, symptoms, hospitalization, ventricular remodeling, and survival, without interpreting surrogate changes as proof of regeneration. Progression requires myocardial-compatible mechanics and degradation, durable spatially controlled electroactivity, chemically defined bioactive release, vascular and tissue integration, acceptable rhythm and blood-contact safety, scalable manufacturing, and Good Manufacturing Practice-compliant quality control [117]. Until these criteria are demonstrated in clinically relevant large-animal studies, PEG/PCL/MXene/ginseng scaffolds should remain a rational preclinical design strategy rather than a clinically validated regenerative therapy.

## 7 Future perspectives and outstanding research gaps

Future studies should determine whether integrated PEG/PCL/MXene/ginseng scaffolds outperform simpler formulations through matched single-component, dual-component, and complete-scaffold comparisons [3], while establishing each component’s measurable, reproducible contribution within a controlled, scalable system. Standardized characterization should track component interactions during degradation and their biological consequences [109]. Key gaps include MXene’s long-term electroactive stability and material fate under post-infarction oxidative and inflammatory conditions [9], as well as bioink rheology, print fidelity, vascular integration, electrical stability, contractile maturation, and durable host integration in bioprinted cardiac constructs; these outcomes must be assessed beyond cell viability and short-term functional gains [58]. Ginseng-derived cargo requires defined composition, dose-response relationships, release profiles, and matrix-compatibility assessment [98].

## 8 Conclusion

Electroactive and bioactive hydrogels may address post-infarction mechanical dysfunction, electrical discontinuity, inflammation, vascular impairment, and fibrosis. PEG/PCL offers tunable processing, mechanics, degradation, and cargo delivery; MXene may provide electroactive interfaces; ginseng-derived bioactives may modulate oxidative, inflammatory, vascular, and fibrotic pathways; and 3D bioprinting can spatially organize these functions. However, component and partial-formulation evidence does not establish efficacy, safety, or synergy of the full PEG/PCL/MXene/ginseng scaffold. Translation requires demonstrated benefit over simpler formulations and conductive alternatives through matched mechanical, electrophysiological, biological, and safety comparisons; durable electroactivity and biosafety; compatible mechanics and degradation; defined ginseng-cargo release; vascular and tissue integration; and compatibility with post-MI pharmacotherapy. Further requirements include validated critical quality attributes, scalable, reproducible manufacturing, batch comparability, chronic large-animal testing, rhythm surveillance, material-fate analysis, and a clear regulatory pathway. The integrated platform remains preclinical, not a validated regenerative therapy.

## Significance

Myocardial infarction (MI) repair requires coordinated mechanical, electrical, inflammatory, fibrotic, and vascular support, yet existing biomaterials address only subsets. This review critically integrates poly(ethylene glycol)/poly(ε-caprolactone) (PEG/PCL) matrices, MXene electroactivity, ginseng-derived bioactives, and 3D bioprinting, comparing conductive materials across performance, stability, biosafety, manufacturability, and electrophysiological function. It distinguishes component-level evidence from the unvalidated integrated platform and identifies key comparative, safety, quality-control, manufacturing, and regulatory requirements for translation.

## CRediT authorship contribution statement

**Azadeh Rejali:** Writing – review & editing, Writing – original draft, Visualization, Validation, Methodology, Data curation, Conceptualization. **Mehdi Ebrahimian-Hosseinabadi:** Writing – review & editing, Validation, Supervision, Conceptualization. **Mohammad Rafienia:** Writing – review & editing, Supervision. **Ghasem Dini:** Writing – review & editing, Supervision.

## Declaration of competing interest

The authors declare that they have no known competing financial interests or personal relationships that could have appeared to influence the work reported in this paper.

## Data Availability

No data was used for the research described in the article.
