# Supplementary material for: Electroactive hydrogel scaffolds for myocardial infarction repair: Integrating MXene, ginseng-derived bioactives, and 3D bioprinting
Source: Biomater Biosyst. 2026 Oct 29;24:100154. doi: 10.1016/j.bbiosy.2026.100154 (PMC13756188; doi:10.1016/j.bbiosy.2026.100154)
Supplement: Supplementary file 1 [file mmc1.docx]

**Supplementary Table S1. Translational maturity, evidence gaps, and regulatory considerations for cardiac biomaterial strategies.**

References [1–117] correspond to the reference list of the main manuscript. Additional references [S1–S15] are cited in the relevant table rows and listed separately below.

| **Strategy / technology** | **Current evidence stage** | **Large-animal evidence** | **Clinical evidence** | **Key limitations and regulatory considerations** | **Ref.** |
| --- | --- | --- | --- | --- | --- |
| Injectable myocardial ECM hydrogel | Porcine preclinical studies; first-in-human feasibility. | Porcine MI: catheter-compatible transendocardial delivery and exploratory remodeling outcomes. | VentriGel: nonrandomized 15-patient study 60 days–3 years post-MI; feasibility and preliminary safety, not established efficacy. | Donor and decellularization controls; sterility, potency, batch release, catheter safety and randomized efficacy testing. | [23, 55] |
| Alginate LV augmentation | Randomized clinical benchmark for ventricular support, not remuscularization. | The clinical surgical program is not a directly comparable large-animal regenerative scaffold study. | AUGMENT-HF (n=78): improved functional capacity in advanced HF; survival benefit not established. | Thoracotomy and procedural risk; persistent implant; limited extrapolation to post-MI injections or epicardial patches. | [24, 108] |
| Conductive hydrogels and cardiac patches | Mostly in vitro and short-term rodent evidence for conductive formulations. | Selected vascularized or drug-delivery patches reached pigs; this does not validate conductive MXene implants. | No established human efficacy for conductive post-MI hydrogel or patch therapy. | Conduction mapping and telemetry; ROS/strain durability; adhesion, material fate, sterilization and scale-up. | [73, 78, 95] [S1] |
| 3D/4D cardiac constructs and EHM benchmark | Bioprinted constructs remain preclinical; EHM allografts are a distinct translational comparator. | EHM allografts studied in rhesus macaques; not direct validation of 3D/4D conductive bioinks. | BioVAT-HF: early open-label EHM data under immunosuppression; neither EHM efficacy nor efficacy of bioprinted patches established. | Cell dose and identity, maturation, perfusion, immune/tumor/rhythm safety, print fidelity, storage and GMP. | [61, 64–66] |
| MXene-enabled cardiac hydrogels and patches | Emerging in vitro and short-term rodent studies; no established human product. | No chronic large-animal MI validation of MXene hydrogels/patches identified among the cited studies. | No clinical safety or efficacy evidence identified for MXene-enabled post-MI therapy. | ROS-driven conductivity loss, flake/etchant variability, dispersion, degradation fate, thrombosis and arrhythmia risks. | [9, 42, 83] [S2, S3] |
| Ginseng-derived bioactive delivery | Pharmacological rationale and early local-hydrogel evidence; formulation-specific validation needed. | No large-animal MI validation identified for a ginseng-loaded cardiac scaffold in the cited studies. | No clinical trial of ginseng-loaded cardiac scaffolds identified; systemic interactions are a separate risk signal. | Defined Panax source and chemical markers; loading, release, potency, systemic exposure, stability and drug interactions. | [103, 104, 106] [S4–S12] |
| Integrated PEG/PCL/MXene/ginseng platform | Proposed preclinical design hypothesis; complete combination untested in MI. | No formulation-specific large-animal evidence identified. | No human evidence; additive benefit, synergy and therapeutic efficacy remain unproven. | Fix product and route; factorial controls, validated CQAs and assays, chronic rhythm/material-fate tests, GMP and regulatory classification. | [91, 112, 117] [S13–S15] |

**Evidence Limitations**: Evidence stages and reported outcomes are specific to the cited formulations and clinical settings. “No evidence identified” indicates that direct evidence was not found among the cited sources; it does not constitute systematic proof of absence. Component-level or comparator findings do not establish the efficacy, safety, or synergistic effects of the proposed integrated PEG/PCL/MXene/ginseng platform.

**Abbreviations**: 3D, three-dimensional; 4D, four-dimensional; AUGMENT-HF, clinical trial of left ventricular augmentation with alginate hydrogel in heart failure; BioVAT-HF, Biological Ventricular Assist Tissue in Terminal Heart Failure; CQA, critical quality attribute; ECM, extracellular matrix; EHM, engineered heart muscle; GMP, good manufacturing practice; HF, heart failure; LV, left ventricular; MI, myocardial infarction; MXene, two-dimensional transition-metal carbides, nitrides, and carbonitrides; PCL, poly(ε-caprolactone); PEG, poly(ethylene glycol); ROS, reactive oxygen species.

Supplementary References:

These additional sources are cited in the Ref. column of Supplementary Table S1; references [1–117] are listed in the main manuscript.

**[S1]** Aboagye J, Edwards M, Ge J, Hong Y, Yang H. Evaluating and improving biocompatibility of conductive polymers for cardiac tissue engineering. J Mater Chem B. 2026;14(3):775–798. doi:10.1039/D5TB01523E.

**[S2]** Guan H, Zhang Y. Applications of elastic and conductive hydrogels in myocardial infarction repair. Colloid Interface Sci Commun. 2025;69:100856. doi:10.1016/j.colcom.2025.100856.

**[S3]** Lv Q, Zhou D, He Y, Xu T, Qiu X, Zeng J. Engineering functional electroconductive hydrogels for targeted therapy in myocardial infarction repair. Bioact Mater. 2025;49:172–192. doi:10.1016/j.bioactmat.2025.01.013.

**[S4]** Ratan ZA, Haidere MF, Hong YH, Park SH, Lee JO, Lee J, Cho JY. Pharmacological potential of ginseng and its major component ginsenosides. J Ginseng Res. 2021;45(2):199–210. doi:10.1016/j.jgr.2020.02.004.

**[S5]** Tang MM, Zhao ST, Li RQ, Hou W. Therapeutic mechanisms of ginseng in coronary heart disease. Front Pharmacol. 2023;14:1271029. doi:10.3389/fphar.2023.1271029.

**[S6]** Zhang XM, Qu SC, Sui DY, Yu XF, Lv ZZ. Effects of ginsenoside-Rb on blood lipid metabolism and anti-oxidation in hyperlipidemia rats. Zhongguo Zhong Yao Za Zhi. 2004;29(11):1085–1088. doi:10.3321/j.issn:1001-5302.2004.11.019.

**[S7]** Shin JH, Kwon HW, Cho HJ, Rhee MH, Park HJ. Vasodilator-stimulated phosphoprotein-phosphorylation by ginsenoside Ro inhibits fibrinogen binding to αIIb/β3 in thrombin-induced human platelets. J Ginseng Res. 2016;40(4):359–365. doi:10.1016/j.jgr.2015.11.003.

**[S8]** Aravinthan A, Kim JH, Antonisamy P, Kang CW, Choi J, Kim NS, Kim JH. Ginseng total saponin attenuates myocardial injury via anti-oxidative and anti-inflammatory properties. J Ginseng Res. 2015;39(3):206–212. doi:10.1016/j.jgr.2014.12.001.

**[S9]** Gou D, Pei X, Wang J, Wang Y, Hu C, Song C, Cui S, Zhou Y. Antiarrhythmic effects of ginsenoside Rg2 on calcium chloride-induced arrhythmias without oral toxicity. J Ginseng Res. 2020;44(5):717–724. doi:10.1016/j.jgr.2019.06.005.

**[S10]** Wang Y, Dong J, Liu P, Lau CW, Gao Z, Zhou D, Tang J, Ng CF, Huang Y. Ginsenoside Rb3 attenuates oxidative stress and preserves endothelial function in renal arteries from hypertensive rats. Br J Pharmacol. 2014;171(13):3171–3181. doi:10.1111/bph.12660.

**[S11]** Zhang N, An X, Lang P, Wang F, Xie Y. Ginsenoside Rd contributes the attenuation of cardiac hypertrophy in vivo and in vitro. Biomed Pharmacother. 2019;109:1016–1023. doi:10.1016/j.biopha.2018.10.081.

**[S12]** Dong H, Ma J, Li T, Xiao Y, Zheng N, Liu J, Gao Y, Shao J, Jia L. Global deregulation of ginseng products may be a safety hazard to warfarin takers: solid evidence of ginseng-warfarin interaction. Sci Rep. 2017;7(1):5813. doi:10.1038/s41598-017-05825-9.

**[S13]** Kamal NH, Heikal LA, Abdallah OY. The future of cardiac repair: a review on cell-free nanotherapies for regenerative myocardial infarction. Drug Deliv Transl Res. 2025;15(7):2253–2271. doi:10.1007/s13346-024-01763-y.

**[S14]** Haghshenas M, Ghazali M, Jannesari M, Dini G, Saki N, Asgarloo S, Abdollahi Asl M. Hybrid conductive polymer nanocomposites: bridging bioelectronics, drug therapy, and regenerative medicine. Results Surf Interfaces. 2026;23:100747. doi:10.1016/j.rsurfi.2026.100747.

**[S15]** Holme S, Richardson SM, Bella J, Pinali C. Hydrogels for cardiac tissue regeneration: current and future developments. Int J Mol Sci. 2025;26(5):2309. doi:10.3390/ijms26052309.
